# Proteinuria and Progression of Renal Damage: The Main Pathogenetic Mechanisms and Pharmacological Approach

**DOI:** 10.3390/medicina60111821

**Published:** 2024-11-06

**Authors:** Elisa Longhitano, Vincenzo Calabrese, Chiara Casuscelli, Silvia Di Carlo, Salvatore Maltese, Adolfo Romeo, Massimo Calanna, Giovanni Conti, Domenico Santoro

**Affiliations:** 1 Unit of Nephrology and Dialysis, Department of Clinical and Experimental Medicine, A.O.U. “G.Martino”, University of Messina, 98125 Messina, Italy; v.calabrese@outlook.it (V.C.); chiara.casuscelli88@gmail.com (C.C.); silviadicarlo1@yahoo.it (S.D.C.); salvatoremaltese389@gmail.com (S.M.); romeo.adolfo@polime.it (A.R.); massimo.calanna@polime.it (M.C.); dsantoro@unime.it (D.S.); 2 Pediatric Nephrology Unit, A.O.U. “G.Martino”, University of Messina, 98125 Messina, Italy

**Keywords:** proteinuria, CKD, ACEi, ARBs, finerenone, sparsentan, SGLT2i, GLP-1

## Abstract

The integrity of the glomerular filtration barrier maintains protein excretion below 150 mg/day. When urinary proteins increase, this indicates damage to the filtration barrier. However, proteinuria is not only a marker of kidney damage but also exacerbates it through various mechanisms involving the glomerular and tubulointerstitial compartments. Therefore, it is essential to intervene with renoprotective action that reduces the proteinuria. In this context, Angiotensin-converting enzyme inhibitors and angiotensin receptor blockers are cornerstone treatments. Recent advancements include sodium–glucose cotransporter 2 inhibitors, initially used for glycemic control, now recognized for their renoprotective properties in both diabetic and non-diabetic populations. Another drug, Finerenone, a selective non-steroidal mineralocorticoid receptor antagonist, has emerged as a promising agent, offering anti-inflammatory and antifibrotic benefits with fewer side effects than traditional steroidal options. Finally, dual inhibition of angiotensin II and endothelin-1 receptors through agents like Sparsentan presents a novel approach with significant antiproteinuric effects in IgA nephropathy and focal segmental glomerulosclerosis. This brief review summarizes the mechanisms by which proteinuria promotes kidney damage and the renoprotective therapeutic approaches available, which can be combined with lifestyle modifications and specific treatments for underlying diseases to mitigate the progression of chronic kidney disease.

## 1. Introduction

The integrity of the glomerular filtration barrier (GFB) allows only low-molecular-weight-proteins and a fraction of albumin (also considered a low-molecular-weight protein in this context) to pass through, which are then both reabsorbed by the proximal tubular cells so that physiologically, the amount of protein in 24 h urine is less than 150 mg. When specific (such as glomerulonephritis) or systemic (such as hypertension and diabetes) damage mechanisms cause dysfunction of the GFB, the urinary share of proteins increases and is referred to as pathological proteinuria.

The diagnosis of proteinuria is important not only as a marker of kidney damage, but mainly because it triggers a series of mechanisms that lead to the progression of the damage itself. One of the first studies to demonstrate this was the Ramipril Efficacy In Nephropathy (REIN) study, which, “collaterally”, showed that patients with proteinuria at baseline had a faster decline in kidney function [1]. The same results were obtained from an aggregate analysis of 11 trials in patients with chronic non-diabetic nephropathy [2]. This analysis showed that the reduction in proteinuria was associated with improved renal outcomes, regardless of the etiology and treatment [2]. Conversely, a worsening of proteinuria was never associated with better renal outcomes [2]. Based on this evidence, research has focused on understanding the mechanisms by which proteinuria leads to the progression of kidney damage.

## 2. Proteinuria and Damage to the Glomerular Compartment

It is now accepted that, regardless of the initial damage, a loss of nephrons exposes the remaining nephrons to adaptive hemodynamic changes aimed at supporting renal function [3].

The glomerular capillary hypertension that results from this compromises the permeability of proteins, which are thus filtered in excessive quantities. The resulting mechanical stress increases the production of angiotensin II (Ang II) and angiotensin AT1 receptors in podocytes, as demonstrated in kidney studies in mice subjected to mechanical strain [4]. The increase in Ang II, regardless of the hemodynamic effects affecting the AT1 receptors, can impair the expression of nephrin, a protein component of the glomerular slit diaphragm [5]. Studies on mice have led to the hypothesis of a mechanism involving activation of the Notch1/HES1/Snail pathway, resulting in subregulation of nephrin, alteration of the slit diaphragm, and increased proteinuria [5]. The increase in Ang II can also stimulate the synthesis of endothelin-1 (End-1), which induces an event similar to the epithelial–mesenchymal transition [6,7].

The excess of proteins in urine to which podocytes are exposed causes stress, which in turn causes phenotypic alterations in podocytes, including the cancellation of foot processes and further dysfunction of the glomerular filtration diaphragm, another event that worsens proteinuria [8]. The loss of podocytes resulting from these processes, in addition to aggravating proteinuria, leads to reduced production of podocyte-derived vascular endothelial growth factor (VEGF) by promoting apoptosis of endothelial cells [9,10]. In addition, the exposure of podocytes to excess protein together with the increase in Ang II stimulate the synthesis of transforming growth factor beta (TGF-β). The latter, on the one hand, contributes to cellular apoptosis and, on the other hand, induces the differentiation of mesangial cells into myofibroblasts, resulting in deposition of the extracellular matrix and glomerulosclerosis [11].

Further, the increase in glomerular permeability causes podocyte dysfunction by altering the mechanism underlying the regeneration of damaged podocytes. Although lost podocytes cannot be replaced, recent studies indicate a potential for differentiation from kidney progenitor cells located within the Bowman [12]. The blocking of the differentiation mechanism is explained by the seizure of retinoic acid by albumin [13].

Finally, the high protein concentration in urine and the subsequent podocyte loss may lead to activation and accumulation of parietal epithelial cells (PEC) within the Bowman’s space as a response to the glomerular lesion, a mechanism involving the complement system [14].

## 3. Proteinuria and Damage to the Tubulo–Interstitial Compartment

Proteinuria also triggers the progression of renal failure through damage to the tubulointerstitial compartment. The renal proximal tubular cells can reabsorb large amounts of albumin through endocytosis, mediated by the megaline and cubiline receptors. Excessive protein absorption at the apical pole of proximal tubular cells is associated with phenotypic changes, including induction of a pro-inflammatory phenotype. The latter leads to glomerular–tubular disconnection with apoptosis of tubular cells. In vivo animal studies have shown that the molecular mechanisms underlying the induction of an inflammatory phenotype in response to proteinuria may be at least partially dependent on the nuclear factor-kappa B (NF-kB) [15,16]. In addition, tubular cells exposed to protein overload activate the apoptotic response. High albumin concentrations, in fact, decrease the megaline expression as it can no longer interact with survival protein kinase B (PKB), which is crucial for the phosphorylation of Bad (death promoter associated with Bcl2) [17]. The lack of iteration does not allow activation of PKB/Akt and phosphorylation of Bad (which would normally inhibit apoptotic response), thus favoring cell apoptosis [17].

Further, the complement plays a crucial role in the tubulointerstitial damage induced by proteinuria. Although the mechanisms are not fully understood, a plausible hypothesis is that the damage results from local synthesis and ultrafiltered C3 [17].

End-1 also plays a key role. Studies have shown that protein overload in urine induces a significant dose-dependent increase in the synthesis of End-1 by proximal tubular cells [18]. End-1, in addition to its vasoactive properties, stimulates macrophage infiltration and promotes interstitial fibroblast proliferation and extracellular matrix synthesis. Increased production of End-1 could mediate tubulointerstitial fibrosis and the progression of kidney disease [18].

Another mechanism involved in tubulointerstitial damage is the activation of resident monocytes and lymphocytes. Kidney interstitium contains numerous resident myelocytes and monocytes, which express dendritic cell markers and may present the antigen. An inflammatory environment converts the tolerogenic state of dendritic cells into an immunogenic state, thus promoting the recruitment of T-cells. It seems that this process may be linked to albumin N-terminal residue that is absorbed by dendritic cells, where it is processed by proteasomes in antigenic peptides. The latter, via the major histocompatibility system, activate CD8+ T cells, which are recruited into the renal interstitium, where they produce Interferon (INF)-y, causing macrophagic activation [17].

## 4. Main Current Pharmacological Approaches for Reducing Proteinuria and Protecting Kidney Function

Studies leading to an understanding of the mechanisms of proteinuria and disease progression mediators in proteinuric nephropathies have improved the effectiveness of renal protection interventions. In particular, therapeutic applications have been concentrated mainly on inhibiting the renin–angiotensin–aldosterone system and secondary pathways of inflammation and fibrosis.

### 4.1. Inhibitors of the Renin–Angiotensin–Aldosterone System (RAAS)

The discovery of the antiproteinuric and nephroprotective effect of angiotensin-converting enzyme inhibitors (ACEis) and angiotensin receptor blockers (ARBs) is a key milestone in nephrology.

We know that the mechanism is partly mediated by the reduction of the tone in the efferent arteriole. This leads to a reduction in the intraglomerular pressure and then in the glomerular stress, as well as to a reduction in the proteinuria, with an initial reduction in the filtrate and subsequent stabilization [19].

ACEis and ARBs effectively reduce proteinuria of any cause by improving renal outcomes [19,20,21,22,23,24,25,26]. Studies in which they were combined have not shown apparent efficacy without risk of adverse outcomes when compared to single use [27,28,29,30]. Based on these findings, the 2024 Kidney Disease Improving Global Outcomes (KDIGO) guidelines recommend starting ACEi or ARB in patients with proteinuria and titrating up to the maximum tolerated dosage [31].

### 4.2. Sodium–Glucose Cotransporter 2 (SGLT2) Inhibitors

Sodium–glucose cotransporter 2 inhibitors (SGLT2i) are drugs introduced to treat hyperglycemia that have shown efficacy in reducing proteinuria. The main mechanism underlying their effect is a reduction in intraglomerular pressure. This results from the blockage of the sodium–glucose cotransporter SGLT2 in the proximal tubule, leading to an increase in sodium and glucose reaching the distal tubule. At the level of dense macula, the increase in sodium triggers tubulo–glomerular feedback, causing vasoconstriction of the afferent glomerular arteriole and vasodilatation of the efferent glomerular arteriole, with a reduction of intraglomerular pressure and improvement of hyperfiltration [32]. Many other routes may also contribute to the beneficial effects of these agents, including diuretic effect, moderate improvements in blood pressure and glycemic control, increased hematocrit, reduction in tubular activity due to reduced sodium resorption resulting in increased oxygen supply to renal tissue, together with reductions in proinflammatory and profibrotic cytokines, and reduction in oxidative stress.

Regarding proteinuria, studies in diabetic and non-diabetic mice with bovine serum albumin-induced damage have shown that dapagliflozin reduced proteinuria, glomerular damage and dysfunction, and podocyte loss [33].

The effect of SGLT2 on proteinuria in humans was highlighted in 2016 by the EMPA-REG OUTCOME trial. This trial showed, as a secondary outcome, a reduction in progression of the albuminuria in the Empagliflozin arm compared to placebo in patients with type 2 diabetes mellitus (T2DM) and estimated glomerular filtration rate (eGFR) ≥ 30 mL/min/1.73 m^2^ of body surface area (Supplementary Table S1) [34]. The following year, the Canagliflozin Cardiovascular Assessment Study (CANVAS) program, which included the Canagliflozin Cardiovascular Assessment Study–Renal (CANVAS–R) study, carried out in diabetic patients, highlighted a potential benefit of Canagliflozin in slowing the progression of albuminuria [35]. A few years later, the Canagliflozin and Renal Events in Diabetes with Established Nephropathy Clinical Evaluation (CREDENCE) study, carried out in diabetic patients with a urinary albumin/creatinine ratio (UACR) > 300 mg/g, showed that canagliflozin reduced the UACR by 31% compared to placebo after 26 weeks of treatment [36].

The Dapagliflozin and Prevention of Adverse Outcomes in chronic kidney disease (DAPA–CKD) study has been the first to include about one-third of patients with non-diabetic kidney disease. The study demonstrated that dapagliflozin, compared to placebo, reduces albuminuria in patients with chronic kidney disease (CKD) with and without diabetes [37]. Particularly in patients with T2DM, the reduction was by 35.1% versus 14.8% in patients without T2DM, compared to placebo [37]. However, these results were not confirmed in the DIAMOND study, which showed that dapagliflozin did not produce any additional reduction in proteinuria levels among patients with CKD who were already receiving RAAS blockers (the difference was 0.9%, *p*-value was 0.93) [38]. The short treatment period (6 weeks) likely influenced this result. The EMPA–KIDNEY trial, after 2 years of treatment, showed that the geometric mean UACR was 19% lower in the empagliflozin group compared to placebo, demonstrating benefits even in non-diabetic patients [39].

Based on the results of these studies, the indication for SGLT2i has expanded, including in non-diabetic CKD patients with albuminuria. The 2023 UK guidelines, in fact, recommend using SGLT2i in people with or without type 2 diabetes with UACR ≥ 200 mg/g and eGFR ≥ 20 mL/min/1.73 m^2^ [40]. Further, the 2024 KDIGO on management of CKD introduced a new recommendation for treating nondiabetic–CKD with eGFR ≥ 20 mL/min and UACR ≥ 200 mg/g with SGLT2i [31].

### 4.3. New Selective Non-Steroidal Mineral Corticoid Receptor Antagonist

Overactivation of mineralocorticoid receptors (MR) may occur in the context of aldosterone excess or in the context of modulation of mineralocorticoid receptor expression secondary to different pathological conditions (such as diabetes, CKD with elevated proteinuria, heart failure, myocardial infarction, hypertension, vascular aging, or cerebral aneurysm) [41].

Overactivation of MR contributes to the progression of CKD and increases proteinuria through inflammation, fibrosis, and damage to podocytes. The antiproteinuric effect of steroidal MR antagonists has long been known, as well as the risk of gynecomastia and hyperkalemia [42]. These latter adverse effects are overcome by the new non-steroidal selective MR antagonist (ns-MRA), finerenone, that has anti-inflammatory and antifibrotic activity in the kidney and heart [43]. As demonstrated by several phase II (ARTS, ARTS–DN, ARTS–HF) and phase III (Finerenone in reducing kidney failure and disease progression in diabetic kidney disease (FIDELIO–DKD) and Finerenone in reducing cardiovascular mortality and morbidity in diabetic kidney disease (FIGARO–DKD) (Supplementary Table S2)) studies, and by the FIDELITY pooled-analysis of patients with type 2 diabetes and CKD, finerenone causes a dose-dependent reduction in albuminuria with side effects similar to placebo and a higher risk of hyperkalemia, but not such that it often leads to the discontinuation of the drug [44,45,46,47,48,49].

Given these findings, finerenone was approved by the Food and Drug Administration (FDA) in 2021 and by the European Medicines Agency (EMA) in 2022 in adults with CKD associated with type 2 diabetes to reduce kidney and cardiovascular adverse events. It is now also available in Italy for the treatment of CKD in stages 3 and 4, associated with type 2 diabetes, in adult patients with albuminuria (30–5000 mg/gr).

Although the studies on finerenone offer significant promise in reducing proteinuria and a good safety profile, most of the results are mainly focused on the short-term and do not provide adequate information on long-term efficacy and safety.

In the FIDELIO–DKD study, the median follow-up period during which a reduced risk of progression of CKD and cardiovascular events was observed compared to placebo was 2.6 years [44]. Similarly, in the FIGARO–DKD trial, with a duration of 3.4 years, treatment with finerenone improved cardiovascular outcomes in patients with CKD with albuminuria [46]. Several clinical trials are currently underway: two observational studies on patients with CKD and T2DM already treated with finerenone (FINEROD and FINE–REAL), a study on kidney transplant patients with albuminuria (EFFEKTPR), and a study on patients with slightly reduced or preserved ejection fraction (REDEFINE–HF). Finally, an ongoing study will answer the question of the efficacy of finerenone in non-diabetic CKD (FIND–CKD). These and future longitudinal studies can provide answers on efficacy and safety in the long-term.

### 4.4. Dual Inhibition of Angiotensin II and Endothelin-1 Receptors

Given the pro-inflammatory and pro-fibrotic action of Ang II and End-1 in the glomerular and tubulointerstitial compartments, sparsentan’s benefit as a reno-protective agent is unsurprising. This new drug simultaneously blocks the activation of the Ang II receptor type 1 (AT1) and the activation of the endothelin type A receptor (ETA). The combined inhibitory action of sparsentan was explored following evidence that adding endothelin receptor antagonists to patients already receiving RAASi reduced residual proteinuria [50,51,52,53,54,55].

Based on the significant antiproteinuric effect demonstrated in the PROTECT trial, the Food and Drug Administration (FDA) has approved sparsentan for the treatment of proteinuria in adult patients with IgA Nephropathy (IgAN) at risk of rapid disease progression (UACR reduction of sparsentan (−42.8%; 95% CI, −49.8% to −35.0%) versus irbesartan (−4.4%; 95% CI, −15.8% to 8.7%)) [56].

Studies on sparsenten are also advancing in patients with focal segmental glomerulosclerosis (FSGS). In the phase II DUET study, a randomized, double-blind with active control study, sparsentan demonstrated superior antiproteinuric efficacy compared to irbesartan over an 8-week treatment period (proteinuria reduction: sparsentan compared with irbesartan (−44.8%; 95% CI, −52.7% to −35.7% vs. −18.5%; 95% CI, −34.6% to 1.7%; *p* = 0.006)) [57]. Another phase III study (DUPLEX) evaluated the long-term antiproteinuric and nephroprotective effects and safety of sparsentan compared to irbesartan in patients with FSGS. The results showed that, at week 108, a more significant percentage of patients in the sparsentan group than in the irbesartan group had partial remission of proteinuria (18.5% vs. 7.5%; relative risk, 2.47; 95% CI, 1.37 to 4.45) [58].

### 4.5. GLP-1 Receptor Agonists

GLP-1 receptor agonists (GLP-1RA) are drugs introduced for the treatment of T2DM. They are known to improve glycemic control and promote weight loss through mechanisms such as incretin release and satiation regulation. Recent interest has focused on their potential renal benefits in patients with T2DM and albuminuria. Clinical trials, such as the Liraglutide Effect and Action in Diabetes: Evaluation of Cardiovascular Outcome Results (LEADER) study and SUSTAIN-6, have shown that liraglutide and semaglutide can reduce urinary albumin excretion and protect kidney function [59,60]. In the LEADER study, treatment with liraglutide significantly decreased macroalbuminuria and the risk of progression to severe kidney disease, with a hazard ratio of 0.78 compared to placebo [59]. In addition, a 22% reduction in the risk of new-onset proteinuria and serum creatinine doubling was observed [59]. The SUSTAIN-6 study similarly showed that semaglutide reduced the risk of new or worsening nephropathy by 36% [60]. These renoprotective effects stem from glycemic control and mechanisms such as reducing oxidative stress, inflammation, and fibrosis.

An aggregate analysis of the two studies revealed a 24% reduction in albuminuria from baseline to 2 years with semaglutide or liraglutide treatment compared to placebo (*p* < 0.001). This suggests that both agents have protective effects on the kidney, with semaglutide 1 mg showing the most pronounced effects.

Based on these promising results, a study (Evaluate Renal Function with Semaglutide once weekly, FLOW) has been specifically designed to assess the effects of GLP-1 on kidney outcomes in people with diabetes and kidney disease. The study showed, among 3533 randomized participants (1767 in the semaglutide group and 1766 in the placebo group), a 40% reduction in the UACR in the semaglutide group compared to a 12% reduction in the placebo group after 104 weeks [61].

## 5. New Drugs and Current Guidelines

The pathological mechanisms underlying proteinuria and the progression of CKD involve hemodynamic alterations, inflammation, and fibrosis. Therefore, a multifaceted therapeutic approach would be desirable. In this regard, the combination of agents such as RAAS inhibitors (RAASi), SGLT2i, GLP-1 RA, and ns-MRA is attracting considerable interest. Emerging evidence from diabetic patients suggests that the combination of multiple drugs is possible. Multitherapy can improve cardiorenal risk more effectively than individual therapies and, in some cases, even improve adverse outcomes. A randomized trial of patients with CKD showed that dual treatment with SGLT2i and eplerenone reduced proteinuria more than monotherapy [62]. The FIDELITY pooled-analysis showed that the beneficial effects of finerenone on cardiac outcomes were independent of the concomitant use of SGLT2i or GLP-1 RA [49]. In addition, the combination of SGLT2i with ns-MRA may offer a safety advantage by mitigating the risk of hyperkalemia. Additional randomized trials, such as the CONFIDENCE study, which evaluates the combined effect of finerenone and empagliflozin in 807 patients with type 2 diabetes and CKD, are critical to providing further insights [63].

The current guidelines, however, already endorse a multifaceted pharmacological approach. The 2022 KDIGO guidelines already recommended inhibition of RAS and SGLT2i as first-line therapy for diabetic patients with CKD, with GLP-1 RA and ns-MRA as subsequent options for enhanced cardiorenal protection [64]. Similarly, the new 2024 guidelines for CKD management recommend dual therapy with RAASi and SGLT2i in non-diabetic patients with albuminuria [31].

## 6. Limitations of Studies

Clinical trials of new nephroprotective drugs have several limitations. On the one hand, long-term results are poor. On the other hand, the limited representativeness of ethnic and geographical diversity makes it difficult to generalize the results, especially in populations with different genetic and socioeconomic profiles from those in the West on which studies are predominantly conducted.

Genetic variants that affect drug metabolism (e.g., variations in the ACE gene or polymorphisms in SGLT2 receptors) could influence patients’ treatment responses. If these variables are not considered, the resulting changes in pharmacokinetics and pharmacodynamics could make drugs less effective or unsafe.

Access to healthcare and the cost of medicines also play a key role in determining the real-world applicability of study results. Low-income populations may encounter barriers to accessing the diagnostic tools needed to monitor chronic conditions and the therapies recommended by clinical guidelines. In fact, despite the cost of downstream complications (such as dialysis), the cost of new drugs often limits their use. Therefore, long-term assessments and more “inclusive” studies that better reflect the heterogeneity of the global population are needed to provide robust and valid evidence for different populations.

## 7. Non-Pharmacological Approaches

**Figure 1:**
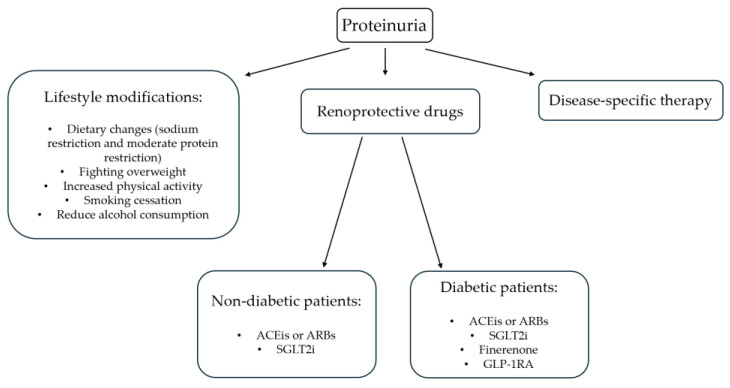
Management of proteinuria. Legend: ACEis, angiotensin-converting enzyme inhibitors; ARBs, angiotensin receptor blockers; SGLT2i, sodium–glucose cotransporter 2 inhibitors; GLP-1RA, GLP-1 receptor agonists.

Lifestyle changes should be considered as complementary to the pharmacological approach in managing patients with proteinuria (Figure 1). “Unhealthy” lifestyles, such as excessive alcohol consumption, sedentary behavior, smoking habits, and obesity, are independently associated with an increased risk of kidney disease [65,66,67,68,69,70,71,72,73]. As for diet, excess protein and salt intake are the two classic risk factors for CKD [74]. The underlying mechanisms may be related to the effect on hyperfiltration, and in this context, some studies have shown that animal proteins have a more damaging impact than plant proteins [74]. The consumption of ultra-processed foods and beverages has also been associated with the risk of incident CKD [75], based on a mechanism that can involve hemodynamic alterations, increased inflammation, and oxidative stress [75]. In general, therefore, a healthy lifestyle could play a central role in the management of the patient with kidney disease. In this context, it is necessary to provide the information and resources patients need to implement the changes, including, for example, referring them to a “renal” nutritionist who knows the benefits and risks associated with different diets in the nephropathic patient.

## 8. Conclusions

Proteinuria is a marker of kidney damage and a cause of kidney disease progression. Its control is essential as a renal protective treatment. RAASi continue to be the most effective drugs for reducing proteinuria. However, new therapeutic options such as SGLT2i have emerged in recent years. At the same time, mineralocorticoid receptors antagonism is gaining importance as a new possibility to reduce residual proteinuria. Integrating these new therapies with specific treatments for the primary disease and lifestyle changes is crucial to preserving kidney function in the long term.

## Supplementary Materials

The following supporting information can be downloaded at: https://www.mdpi.com/article/10.3390/medicina60111821/s1, Table S1: Main Clinical Trials evaluating the effect of SGLT2i on proteinuria; Table S2: Main Clinical Trials evaluating the effect of finerenone on proteinuria.
