# Supplementary material for: Proteinuria and Progression of Renal Damage: The Main Pathogenetic Mechanisms and Pharmacological Approach
Source: Medicina (Kaunas). 2024 Nov 6;60(11):1821. doi: 10.3390/medicina60111821 (PMC11596299; doi:10.3390/medicina60111821)
Supplement: Supplementary file 1 [file medicina-60-01821-s001.zip › medicina-3226871-supplementary.pdf]

Supplementary tables

Supplementary table S1: Main Clinical Trial evaluating the effect of SGLT2i on proteinuria

Study	Type of study	Main entry criteria	Treatment arms	Kidney Outcomes	Proteinuria results
EMPA-REG OUTCOME study (62)	Multicenter, international, randomized, parallel group, double-blind study	Type 2 DM;<br><br>Drug-naïve or pre-treated with any background therapy;<br><br>7%≤HbA1c≤10% for patients on background therapy or 7%≤HbA1c≤9% for patients drug-naïve;<br><br>Age≥18yrs;<br><br>BMI≤45kg/m² at first visit;<br><br>eGFR≥30ml/min/1,73m²;<br><br>High CV risk.	1:1:1<br><br>Empagliflozin 10mg<br><br>Empagliflozin 25 mg<br><br>Placebo	Incident or worsening nephropathy°<br><br>Incident albuminuria	Progression to macroalbuminuria occurred in 11.2% in the empagliflozin group and in 16.2% in the placebo group (a significant relative risk reduction of 38%).<br><br>Incident albuminuria occurred in 51.5% in the empagliflozin group and in 51.2% in the placebo group.
CANVAS Program (CANVAS study + CANVAS-R study) (63)	Two Randomized, multicenter, double-blind studies	Type 2 DM;<br><br>7%≤HbA1c≤10.5%;<br><br>Age≥30yrs with history of CV events or age≥50yrs with high risk of CV events;<br><br>Must be either not on AHA therapy, or on AHA monotherapy, or combination AHA therapy with any approved agent for the control of blood glucose levels.	CANVAS<br>1:1:1<br>Canagliflozin 100mg<br>Canagliflozin 300 mg<br>Placebo<br><br>CANVAS-R<br>1:1<br>Canagliflozin 100 mg/ Canagliflozin 300 mg (initial dose of 100 mg daily with an optional increase to 300 mg starting from week 13)<br><br>Placebo	Progression of albuminuria§	Progression of albuminuria occurred less frequently among participants assigned to canagliflozin than among those assigned to placebo (hazard ratio of 0.73 (95% CI, 0.67 to 0.79);<br><br>Regression of albuminuria also occurred more frequently among those assigned to canagliflozin than among those assigned to placebo (hazard ratio, 1.70; 95% CI, 1.51 to 1.91).
		Age ≥30 yrs;			

CREDENCE study (64)	Randomized, Double-blind, event-driven, placebo-controlled, multicenter study	Type 2 DM;<br><br>6.5%≤ HbA1c≤12.0%;<br><br>30ml/min/1.73m²≤eGFR≤90 ml/min/1.73 m²;<br><br>300<UACR≤5000 mg/g;<br><br>ACEis or ARBs on maximum tolerated doses for at least 4 weeks before randomization.	100 mg canagliflozin daily<br><br>placebo	Primary Composite Endpoint of Doubling of Serum Creatinine*, ESKD°°, and Renal or Cardiovascular (CV) Death§§	The geometric mean of the UACR was lower by 31% (95% CI, 26 to 35) on average during follow-up in the canagliflozin group.
DAPA-CKD study (65)	International, multicenter, event-driven, randomized, double blind, parallel group, placebo-controlled study	Age≥18 yrs;<br><br>25ml/min/1.73m²≤ eGFR≤75ml/min/1.73m²;<br><br>Evidence of increased albuminuria 3 months or more before visit 1 and UACR ≥200 and ≤5000 mg/g at visit 1;<br><br>Stable, and for the patient maximum tolerated labelled daily dose, treatment with ACE-I or ARB for at least 4 weeks before visit 1, if not medically contraindicated	Dapagliflozin 10 mg or 5 mg<br><br>Placebo	Time to the First Occurrence of Any of the Components of the Composite: ≥50% Sustained Decline in eGFR or Reaching ESRD or CV Death or Renal Death;<br><br>Prespecified exploratory outcome: change in albuminuria;<br><br>Additional discrete endpoints: regression in UACR stage** and	Dapagliflozin reduced geometric mean UACR by 29·3% (95% CI -33·1 to -25·2; p<0·0001); particularly in patient with type 2 DM treatment with dapagliflozin resulted in a geometric mean percentage change of -35·1% (95% CI -39·4 to -30·6; p<0·0001) and -14·8% (-22·9 to -5·9; p=0·0016) in patients without type 2 DM over the follow-up visits (pinteraction<0·0001)<br><br>In patients with UACR of 300 mg/g or greater at baseline, dapagliflozin increased the likelihood of regression in UACR stage (hazard ratio 1·81, 95% CI 1·60 to 2·05)

				progression in UACR stage°°° .	In patients with UACR less than 3000 mg/g at baseline, dapagliflozin decreased the risk of progression in UACR stage (hazard ratio 0·41, 0·32 to 0·52)
DIAMOND study(66)	Randomized, double blind 6-weeks, cross-over study	18yrs≤age≤75yrs;<br><br>500mg/g<proteinuria≤3500mg/g in a 24h urine collection;<br><br>eGFR≥25ml/min/1.73m²;<br><br>Stable dose of an ACEi or ARB for at least 4 weeks prior to randomization;	Dapagliflozin 10mg<br><br>Placebo	Change in basaline 24hr proteinuria;<br><br>Effect on mGFR (using iohexol clearance);<br><br>Prespecified exploratory outcomes: 24-h albuminuria, 24-h protein-to-creatinine ratio, and 24-h UACR	Geometric mean 24-h proteinuria decreased by 15·8% (3·5 to 26·4) after 6 weeks in placebo arm and by 15·0% (2·8 to 25·7) after 6 weeks in dapagliflozin arm.<br><br>The difference in mean percentage change in 24-h albuminuria between dapagliflozin and placebo treatment was –11·8% (–30·4 to 12·0; p=0·30;).<br><br>The difference in mean percentage change in proteinto-creatinine ratio between dapagliflozin and placebo treatment was –6·1% (–21·7 to 12·5; p=0·49;).<br><br>The difference in mean percentage change in UACR between dapagliflozin and placebo treatment was –17·0% (–33·2 to 3·4; p=0·095;).

EMPA-KIDNEY study (67)	Multicenter, International, Randomized, Parallel Group, Double-blind, Placebo-controlled clinical study	Age ≥18 yrs or at "full age" as required by local regulation;<br><br>Evidence of CKD at risk of kidney disease progression§§§<br><br>Clinically appropriate doses of ACEi or ARB unless such treatment is either not tolerated or not indicated	Empagliflozin 10 mg<br><br>Placebo	Time to First Occurrence of Kidney Disease Progression***	The geometric mean UACR was 19% lower in the empagliflozin group than in the placebo group (95% CI, 15 to 23)

Legend: DM, diabetes mellitus; HbA1c, hemoglobin A1c; BMI, body mass index; eGFR, estimated glomerular filtration rate; CV, cardiovascular; AHA, antihyperglycemic agents; UACR, Urine Albumin-Creatinine Ratio; ACEis, Angiotensin-Converting Enzyme inhibitors; ARBs, Angiotensin Receptor Blockers; mGFR, misured glomerular filtration rate; CKD, chronic kidney disease;
°defined as progression to macroalbuminuria, doubling of the serum creatinine level, initiation of renal-replacement therapy, or death from renal disease;
§defined as more than a 30% increase in albuminuria and a change from either normoalbuminuria to microalbuminuria or macroalbuminuria or from microalbuminuria to macroalbuminuria;
*from baseline average determination (sustained and confirmed by repeat central laboratory measure after at least 30 days and preferably within 60 days);
°°defined as initiation of maintenance dialysis for at least 30 days, or renal transplantation, eGFR<15mL/min/1.73 m2 (sustained and confirmed by repeat central laboratory measure after at least 30 days and preferably within 60 days);
§§renal death defined as death in participants who had reached ESKD, died without initiating renal replacement therapy, and no other cause of death was determined via adjudication);
**defined as a transition from macroalbuminuria (≥300 mg/g) to microalbuminuria or normoalbuminuria (<300 mg/g);
°°°defined as a transition from less than 3000 mg/g to 3000 mg/g or greater;
§§§ defined by at least 3 months before and at the time of screening visit
20ml/min/1.73m^2≤eGFR<45ml/min/1.73m^2
or 45ml/min/1.73m^2≤eGFR<90ml/min/1.73m^2 with UACR≥200 mg/g (or protein:creatinine ratio ≥300 mg/g);
***definite as ESRD (dialysis or kidney transplant) or sustained decline in eGFR to <10 mL/min/1.73m or renal death or sustained decline of ≥40% in eGFR from randomization;

Supplementary table S2: Main Clinical Trial evaluating the effect of finerenone on proteinuria

Study	Type of study	Main entry criteria	Treatment arms	Kidney Outcomes	Proteinuria results
	A Randomized, Double-blind, Placebo-controlled, Parallel-group, Multicenter, Event-	Age≥18 yrs;<br><br>Type 2 DM;	Finerenone 10 mg or 20 mg<br><br>Placebo	The First Occurrence of the Composite Endpoint of Onset of	

FIDELIO-DKD study (72)	driven Phase 3 Study	CKD with at least one of: 30≤UACR<300mg/g in 2 out of 3 first morning void samples and estimated 25≤eGFR<60 mL/min/1.73 m² and presence of diabetic retinopathy or UACR ≥300 mg/g in 2 out of 3 first morning void samples and 25≤eGFR<75 mL/min/1.73 m²<br><br>Prior treatment with ACEis or ARBs or both for at least 4 weeks before the run-in visit and from the run-in visit onward only ACEi or ARB. Additionally, for at least four weeks before the screening visit, subjects should be on the highest tolerated dose of either an ACEI or ARB, with no changes to their medication or other related treatments.<br><br>Serum potassium ≤4.8 mmol/L at both the run-in and the screening visit		Kidney Failure, a Sustained Decrease of eGFR ≥40% From Baseline Over at Least 4 Weeks, or Renal Death;<br><br>Change in UACR from Baseline to month 4;<br><br>The First Occurrence of the Composite Endpoint of Onset of Kidney Failure, a Sustained Decrease in eGFR of ≥57% From Baseline Over at Least 4 Weeks, or Renal Death	Finerenone was associated with a 31% greater reduction in the UACR from baseline to month 4 than placebo (ratio of least-squares mean change from baseline [finerenone vs. placebo], 0.69; 95% CI, 0.66 to 0.71).
FIGARO-DKD (74)	A Randomized, Double-blind, Placebo-controlled, Parallel-group,	Age≥18 yrs;<br><br>Type 2 DM;<br><br>DKD with persistent high albuminuria or	Finerenone 10 mg or 20 mg<br><br>Placebo	The First Occurrence of the Composite Endpoint of Onset of Kidney Failure, a	

	Multicenter, Event-driven Phase 3 Study	persistent very high albuminuria at the Run-In and Screening Visit Pretreated with either ACEi or ARB at maximal tolerated labeled dose without adjustments;<br><br>Serum potassium <=4.8 mmol/L		Sustained Decrease of eGFR ≥40% From Baseline Over at Least 4 Weeks, or Renal Death;<br><br>Change in UACR from baseline to month 4;<br><br>The First Occurrence of the Composite Endpoint of Onset of Kidney Failure, a Sustained Decrease in eGFR of ≥57% From Baseline Over at Least 4 Weeks, or Renal Death	The reduction in the UACR from baseline to month 4 was 32% greater with finerenone than with placebo (ratio of the least-squares mean change from baseline, 0.68; 95% CI, 0.65 to 0.70)

Legend: DM, diabetes mellitus; CKD, chronic kidney disease; UACR, urine albumin-to-creatinine ratio; eGFR, estimated glomerular filtration rate; ACEis, Angiotensin-Converting Enzyme inhibitors; ARBs, Angiotensin Receptor Blockers; DKD, diabetic kidney disease;
